# Efficacy of wIRA in the treatment of sacroiliitis in male patients with ankylosing spondylitis and its effect on serum VEGF levels

**DOI:** 10.1186/s13018-019-1322-7

**Published:** 2019-09-18

**Authors:** Jian Xu, Yao Deng, Chun-Yan Yu, Zhao-Meng Gao, Xi-Rui Yang, Qi Zhang, Lei Zhang

**Affiliations:** Department of Rheumatology and Immunology, Shengli Oilfield Central Hospital, No. 31 of Jinan Road, Dongying, 257034 Shandong China

**Keywords:** Spondylitis, Ankylosing, Sacroiliitis, Ultrasound, Water-filtered infrared-A (wIRA), Physical therapy modality

## Abstract

**Background:**

This study aimed to assess the efficacy of water-filtered infrared A (wIRA) in sacroiliitis in male patients with ankylosing spondylitis (AS) and the effect of wIRA therapy on serum vascular endothelial growth factor (VEGF).

**Methods:**

One hundred twenty male AS patients with active sacroiliitis were randomly divided into wIRA group and control group. wIRA treatment was performed twice daily for 5 consecutive days with 24-h interval before switching the treatment (crossover design). Bath ankylosing spondylitis disease activity index (BASDAI) scores, pain visual analogue scale (VAS), and morning stiffness VAS were recorded prior to and after each treatment period. Additionally, C-reactive protein (CRP), serum VEGF, and resistance index (RI) of sacroiliac joints detected by ultrasonography were recorded at baseline and after the first and second treatment period, respectively. The efficacy was examined by using repeated measures analysis of variance (ANOVA).

**Results:**

BASDAI, pain VAS, and morning stiffness VAS scores decreased significantly (*P* < 0.001) after wIRA treatment and no-wIRA treatment (control group), and the difference between the two groups was significant (*P* < 0.001). CRP declined and RI increased during the wIRA treatment as compared with the no-wIRA treatment (*P* < 0.001). The increase in RI was associated with improvement of pain VAS scores (*P* = 0.018), while serum VEGF was unaffected by the treatment.

**Conclusions:**

wIRA treatment achieved symptom and pain relief for AS patients with active sacroiliitis. wIRA treatment also improved RI revealed by ultrasonography, and this effect was associated with improved pain VAS scores.

## Introduction

Ankylosing spondylitis (AS), associated with human leukocyte antigen B27, is a chronic immune disorder mainly affecting the axial skeleton, which predominantly affects the young and middle-aged males. Its pathogenesis is largely unknown. AS first invades the synovial membrane of the sacroiliac joint (SIJ). As the disease progresses, articular cartilage and bone are destroyed, and osseous ankylosis and deformity of the spine or peripheral joints, or even disability are finally developed [1]. Early diagnosis and treatment of sacroiliitis are important for improving clinical outcomes. In 1992, Maugars et al. [2] began to treat sacroiliitis by hormone injection into the SIJ, after which some domestic reports have been published in this field. But due to limited conditions, such treatment for sacroiliitis is not extensively utilized. Water-filtered infrared-A (wIRA) treatment represents the latest achievement in medical immunology science. This treatment targets the deep subcutaneous tissues, alleviating local inflammation and benefiting skin trauma, ulcers, burns, and bedsores [3]. However, wIRA treatment has been rarely applied to sacroiliitis in AS. The present study aimed to evaluate the efficacy of wIRA in AS treatment.

## Materials and methods

### Diagnostic and exclusion criteria

Male patients treated at the Department of Rheumatology from December 2015 to February 2018 who met the following inclusion criteria were recruited: aged > 16, conforming to the 1984 Modified New York Criteria for AS [4], and currently in the active stage. The qualifying features of AS were as follows: (1) morning stiffness visual analogue scale (VAS) scores ≥ 3 and (2) VAS scores ≥ 3 in at least two of the items below: ① patient overall scores, ② back pain in the nighttime and overall back pain, and ③ bath ankylosing spondylitis disease activity index (BASDAI). The patients were combined with at least one active inflammatory lesion in the SIJ confirmed by magnetic resonance imaging (MRI) [5]. A total of 120 cases were recruited, with a course of 6.83 ± 3.2 years. They were aged 17–61 years old (average, 32.1 ± 10.5 years). All patients received methotrexate (MTX, 7.5–20 mg/week) and celecoxib (200 mg/day). Exclusion criteria were as follows: (1) fever; (2) uncontrolled hypertension; (3) diabetes; (4) skin bursts at the affected site; (5) severe liver, kidney, and cardiac insufficiency; (6) active gastrointestinal ulcers in the past 3 months and gastrointestinal bleeding in the past 6 months; (7) biologic therapy in the past 3 months; (8) interstitial pulmonary fibrosis; and (9) other conditions deemed unsuitable for the research.

The informed consent was signed by all subjects. The protocol was approved by the Ethics Committee of Shengli Oilfield Central Hospital (No. Q/ZXYY-ZY-YWB-LL201515).

### Study design

The randomized controlled cross-over design was adopted in this study. Using a random number table and remainder method, the subjects were divided into group A (wIRA treatment) and group B (no-wIRA treatment), with 60 subjects in each group. Patients in group A first received wIRA treatment twice daily, 20 min each time for 5 consecutive days. Each treatment cycle lasted for 5 days, and the first stage was finished after the first cycle, followed by 24-h washout period before the next cycle. The second stage was no-wIRA treatment, which was finished 5 days later. Group B received no-wIRA treatment for 5 days, followed by 24-h washout period before the next cycle. The second stage was wIRA treatment twice daily, for 20 min each time for 5 consecutive days.

C-reactive protein (CRP), serum vascular endothelial growth factor (VEGF), and resistance index (RI) of sacroiliac joints detected by ultrasonography were recorded at baseline and after the first and the second treatment period, respectively.

### Ultrasonography of SIJ

Siemens S2000 Ultrasound System with 14L5 transducer (probe frequency 7–14 MHz, total gain 50 dB, color Doppler gain 80–90 dB, dynamic range 50–60 dB, and wall filter 30–40 Hz) was used. The subjects took a prone position, and an appropriate frequency was chosen for the transducer, which was placed at the level of posterior superior iliac spine. First, the iliac bone, sacrum, and first sacral foramina were located on one side by two-dimensional ultrasonography. The upper part of SIJ was scanned slightly laterally and obliquely at the level of the first sacral foramina, and then transversely and obliquely towards caudal. The position of the SIJ was determined along with the manifestations upon two-dimensional ultrasonography. Next, following a reverse path, color Doppler ultrasonography was applied to the blood flow inside and on the periphery of the SIJ. The cross section with the most abundant blood flow signals was chosen for the measurements of RI, and the mean value of the three measurements was taken.

### Disease and clinical symptom evaluation

BASDAI was composed of six items concerning AS-related symptoms, such as fatigue, spinal pain, joint swelling, local tenderness, and morning stiffness, which were measured using a 0–10 scale. The higher the score, the higher the disease activity [6]. Pain VAS scores and morning stiffness VAS scores were also measured on a 0–10 scale, and the higher the score, the higher the activity.

### Serum collection and measurements

From each subject, about 3 ml of the fasting venous blood was collected, and the serum was isolated and cryopreserved at − 80 °C. ELISA kit (Wuhan Boster Bioengineering Limited Company) was used to determine the serum level of VEGF in strict accordance with the instructions. CRP levels were determined by immunoturbidimetric assay.

### wIRA treatment device

Hydrosun™ 750H treatment device (product No. 15700081; power 230 V-50/60Hz, 775 W), imported from Germany by Beijing Haite Technology Co., Ltd., was used. The wIRA light source was placed at about 20–30 cm from the SIJ, and the temperature was adjusted according to the patients’ sensation to avoid burn.

### Statistical analysis

SPSS 19.0 software was used for statistical analysis. Continuous variables were expressed as *x* ± *s*, and categorical variables as percentages. Measurements obeying to a normal distribution and homogeneity of variance were analyzed by *t* test. Otherwise, the measurements were analyzed by Mann-Whitney *U* test. The influence factors between and within the groups and their interactions were analyzed by repeated ANOVA measures. The efficacy before and after different treatment regimens was compared by paired *t* test (Bonferroni correction, *P* value × 2). Ordered multi-class data were compared by Kruskal-Wallis H test, and correlation between two categorical variables was examined by *χ*^2^ test. *P* < 0.05 indicated a significant difference.

## Results

### Baseline information

A total of 120 cases were finally recruited. At the baseline, there was no significant difference in age, course of disease, BASDAI, CRP, VEGF, pain VAS, morning stiffness VAS, and positive rates of HLA-B27 between the two groups (*P* > 0.05). See Table 1.

**Table 1:** Baseline information of the patients

	Group A	Group B	*t*/*x*^*2*^-statistic	*P* value
Age (years)	32.05 ± 10.96	32.60 ± 13.01	− 0.145	0.886
Disease course (years)	6.92 ± 3.21	6.40 ± 2.70	0.554	0.583
HLA-27 positive rates	95% (57/60)	100% (60/60)	1.026	0.311
CRP (mg/L)	30.21 ± 6.98	30.04 ± 8.33	0.070	0.945
VEGF (pg/ml)	180.22 ± 18.51	182.87 ± 23.31	− 0.399	0.692
BASDAI scores (0–10)	8.30 ± 0.78	8.43 ± 0.80	− 0.522	0.604
Pain VAS scores (0–10)	8.90 ± 0.52	8.80 ± 0.54	0.597	0.554
Morning stiffness-VAS scores (0–10)	8.79 ± 0.55	8.77 ± 0.53	0.145	0.885
RI	0.52 ± 0.04	0.54 ± 0.06	− 1.17	0.249

*HLA-27* human leukocyte antigen B27, *CRP* C-reactive protein, *VEGF* vascular endothelial growth factor, *BASDAI* bath ankylosing spondylitis disease activity index, *RI* resistance index, *VAS* visual analogue scale

### Changes of BASDAI, pain VAS scores, and morning stiffness VAS scores

All patients receiving wIRA treatment had a reduction in BASDAI, pain VAS scores, and morning stiffness VAS scores than before (*P* < 0.001), which were also significantly different from those of the control group (no-wIRA treatment) (*P* < 0.001). Different treatment regimens had no impact on BASDAI, pain VAS scores, and morning stiffness VAS scores. At the end of treatment regimen A, these three scores (7.82 ± 0.67; 7.39 ± 0.57; 7.38 ± 0.51, respectively) were not significantly different from those at the end of treatment regimen B (7.83 ± 0.50; 7.19 ± 0.57; 7.14 ± 0.51, respectively), except that they were much lower than the baseline (*P* < 0.001). See Table 2.

**Table 2:** Changes of BASDAI, pain VAS scores, and Morning stiffness-VAS scores

	wIRA group (*n* = 120)	Control group (*n* = 120)	Difference *d*	*t*-statistic	*P* value	95% CI
BASDAI						
Before T^*^	8.33 ± 0.71	8.36 ± 0.74	− 0.04 ± 0.35	− 0.632	0.531	− 0.15~0.08
After T	7.65 ± 0.65	7.90 ± 0.62	− 0.26 ± 0.56	− 2.907	0.000	− 0.44~− 0.08
Difference *d*	0.68 ± 0.46	0.45 ± 0.49	–	0.35 ± 0.50	–	–
95% CI	0.53~0.83	0.30~0.61	–	0.24–0.47	–	–
*P* value	0.000	0.000	–	0.000	–	–
Pain VAS scores						
Before T	8.85 ± 0.48	8.82 ± 0.45	0.03 ± 0.34	0.605	0.549	− 0.08~0.14
After T	7.30 ± 0.65	7.77 ± 0.46	− 0.46 ± 0.54	− 5.436	0.000	− 0.63~− 0.29
Difference *d*	1.55 ± 0.49	1.05 ± 0.35	–	0.27 ± 0.48	–	–
95% CI	1.39~1.70	0.94~1.16	–	0.16–0.37	–	–
*P* value	0.000	0.000	–	0.000	–	–
Morning stiffness VAS scores						
Before T	8.81 ± 0.46	8.75 ± 0.50	0.06 ± 0.37	1.071	0.291	− 0.06~0.18
After T	7.26 ± 0.59	7.80 ± 0.55	− 0.54 ± 0.55	− 6.205	0.000	− 0.72~− 0.36
Difference *d*	1.55 ± 0.50	0.95 ± 0.35	–	0.28 ± 0.50	–	–
95% CI	1.38~1.71	0.83~1.06	–	0.17–0.39	–	–
*P* value	0.000	0.000	–	0.000	–	–

*T* treatment, *BASDAI* bath ankylosing spondylitis disease activity index, *VAS* visual analogue scale, *CI* confidence interval

*Before treatment: wIRA treatment (before the start of the first stage of treatment regimen A and before the start of the second stage of treatment regimen A); control group (before the start of the first stage of treatment regimen B and before the start of the second stage of treatment regimen A). After treatment: values before the corresponding treatment regimen and after the corresponding treatment regimen

### Changes of CRP, VEGF, and RI

**Fig. 1:**
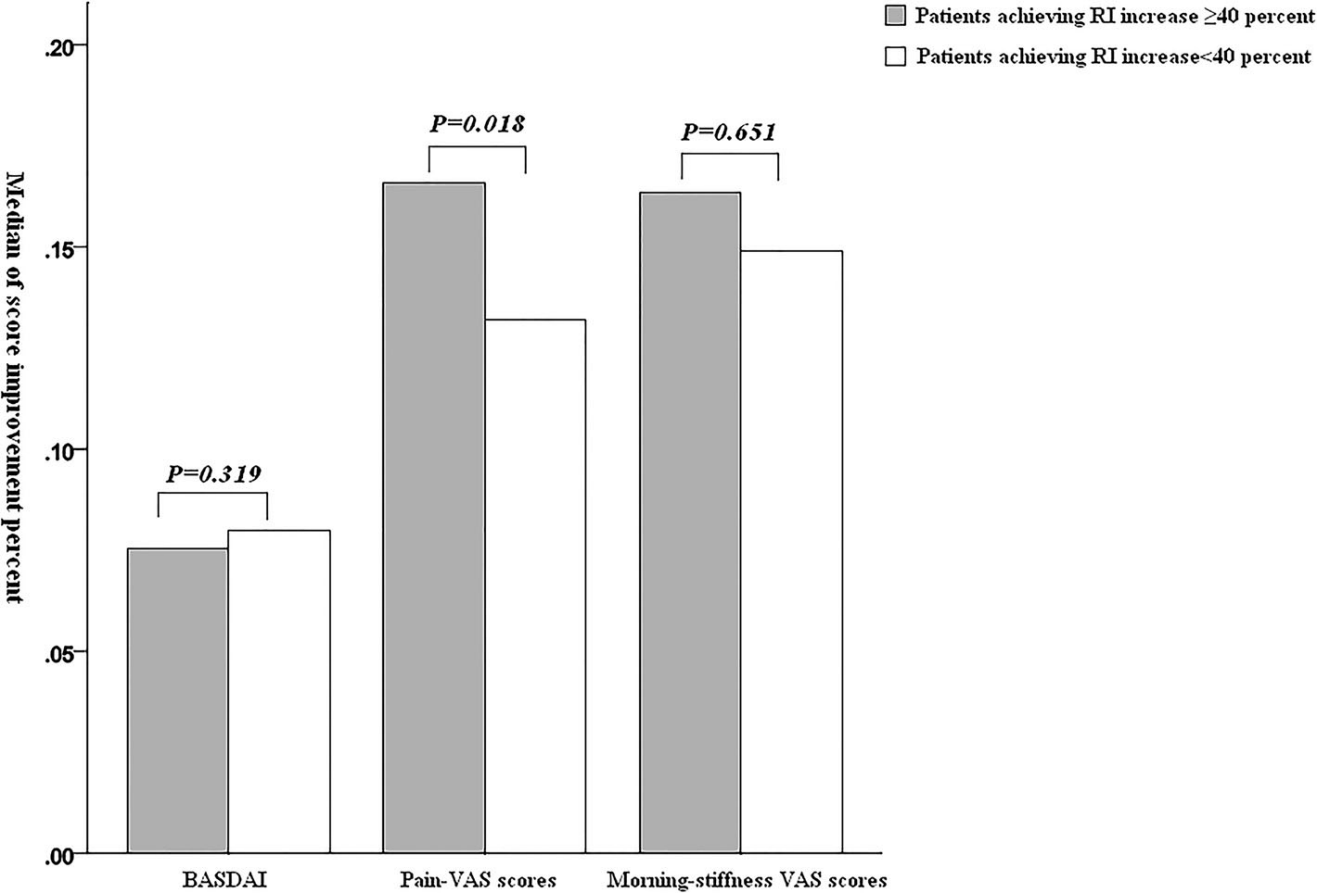
Influence of RI changes on BASDAI, pain VAS scores, and morning stiffness VAS scores. Among all patients receiving wIRA treatment, the improvement of pain VAS scores was greater in those with ≥ 40% increase in RI than in those with < 40% increase in RI. However, there was no significant difference in BASDAI and morning stiffness VAS scores

CRP levels decreased after the end of the first stage of treatment regimen A (receiving wIRA treatment) (*P* < 0.001), and it began to increase after the second stage (*P* = 0.032). The VEGF level slightly decreased after wIRA treatment in either regimen, but there was no significant difference than before treatment (*P* = 0.230 and *P* = 0.120). RI showed an apparent increase at the end of the first stage of treatment regimen A (*P* < 0.001), and it declined further after the end of the second stage (*P* < 0.001), but remained higher than the baseline (*P* < 0.001). See Table 3. Among all patients receiving wIRA treatment, the improvement of pain VAS scores was greater in those with ≥ 40% increase in RI than in those with < 40% increase in RI, and the difference was of statistical significance (*P* = 0.018). However, there was no significant difference in BASDAI and morning stiffness VAS scores (*P* = 0.319, *P* = 0.651, respectively). See Fig. 1.

**Table 3:** Changes of CRP, VEGF and RI

	Before the first stage	After the first stage	After the second stage
CRP (mg/L)			
Treatment regimen A	30.21 ± 6.98	27.33 ± 6.23^a^	29.96 ± 6.21
Treatment regimen B	30.03 ± 8.33	29.55 ± 7.71	26.83 ± 6.08
VEGF (pg/ml)			
Treatment regimen A	180.22 ± 18.51	178.32 ± 16.10^b^	179.63 ± 17.59
Treatment regimen B	182.87 ± 23.31	183.12 ± 21.40^c^	180.45 ± 20.49
RI (0~1)			
Treatment regimen A	0.53 ± 0.05	0.85 ± 0.04^d^	0.63 ± 0.04
Treatment regimen B	0.54 ± 0.05	0.55 ± 0.06	0.83 ± 0.04

*CRP* C-reactive protein, *VEGF* vascular endothelial growth factor, *RI* resistance index

^a^Compared with that before the first stage (*P* = 0.032)

^b^Compared with that before the first stage (*P* = 0.230)

^c^Compared with that before the first stage (*P* = 0.120)

^d^Compared with that before the first stage (*P* < 0.001)

## Discussion

AS is featured by axial skeleton invasion and progressive spine rigidity as well as early involvement of the SIJ. The pathology of sacroiliitis is associated with extensive infiltration of inflammatory cells, such as CD4+ and CD8+ T cells and macrophages. Tumor necrosis factor-α (TNF-α), secreted by the activated T-lymphocytes, can promote the secretion of VEGF by peripheral blood monocytes and synovial cells. VEGF, acting on the osteoblasts, activates the osteoblasts and promotes the osteogenic effect. Several physical and chemical factors at the inflammatory sites are conducive to the expression and secretion of VEGF [7]. Liu et al. [8] found an upregulation of VEGF mRNA in the synovial tissues of AS patients, which was associated with increased BASDAI scores. This indicated that VEGF may directly participate in the differentiation of fibroblasts at the inflammatory sites into osteoclasts. Qian et al. [9] reported a positive expression of VEGF in the synovial tissues of the SIJ in AS patients at the active stage. Moreover, the count of positive cells and mean gray value differed significantly as compared with the control group. This offered evidence that VEGF is closely related to cartilage and bone destruction and bone sclerosis at the SIJ. In addition, hypoxia within the inflammatory joint is very likely to induce an upregulation of hypoxia-inducible factor-1α (HIF-1α), which further leads to the expressions of VEGF and its receptor [10]. Multiple mechanisms may be implicated in the hypoxia within the SIJ. For example, following the bone destruction of SIJ, a large amount of granulation tissues will be formed as a compensatory mechanism, thus affecting local circulation. Inflammatory stimulation causes significant intraarticular synovial hyperplasia, finally resulting in hypoxia of the articular joint and synovium due to hypermetabolism of the newly formed synovium and granulation tissues. In the meantime, the balance of angiogenetic regulation is disrupted by long-term inflammatory stimulation, with local appearance of abnormal pannus. The isolation effect provided by the pannus between the synovium and joint cartilage will also lead to local hypoxia. VEGF is upregulated directly as a result of hypoxia, or through activation of osteoblasts near the sites of bone damage. Before formation of new cartilage, vascular invasion first occurs, and osteoblasts are transported to the sites of ossification and activated. The activated osteoblasts further cause an upregulation of VEGF. Hypoxia and VEGF interact with each other, leading to formation of new bones and finally progressive SIJ fusion and ossification of the ligament [11].

VEGF has been found as an important proangiogenic factor participating in the occurrence and development of arthritis [12]. In the inflammatory SIJ at an active stage, a large amount of VEGF is expressed, which further leads to excessive angiogenesis [13], with local release of inflammatory factors. As a result, there will be a vasodilatation and an increase in blood perfusion at the lesions. An enhancement is usually observed at the lesions revealed by MRI, and there are more abundant blood flow signals revealed by color Doppler ultrasonography, with a reduction in arterial RI. Zhu et al. [14] reported that the angiogenesis of SIJ coincided with the low resistance signals and activity of AS. There was no healthy control group in our study, and we observed an increase of baseline VEGF [(181.54 ± 20.82) pg/ml] and reduction of RI (0.53 ± 0.03) in AS patients, with a detection rate of 90%. This agreed with the previous studies [15–17]. After the wIRA treatment, the RI value of SIJ was significantly increased in AS patients, indicating the weakening of local blood flow signals and attenuation of inflammation. The pain VAS score was also improved significantly, especially in those achieving an increase in RI by over 40%. The above findings on efficacy of wIRA treatment guide the individualized wIRA treatment, but more controlled trials with a large sample size are needed to optimize the intensity and course of treatment.

Sacroiliitis is an early clinical manifestation of AS, and most patients report lower back pain in the nighttime and (or) progressive spine rigidity and deformity. It has been reported [18] that active inflammation of AS is closely related to patients’ life quality and that an effective treatment modality of sacroiliitis is important for improving the outcome. Back in 1992, some scholars [2, 19–21] began to perform CT- or MRI-guided SIJ intra-articular injection of glucocorticoids to treat serum-negative spondyloarthropathy, which were effective in attenuating SIJ inflammation. However, this treatment modality is invasive and places a higher requirement on physician’s skills, contributing to a low acceptance among the patients [22]. Alternatively, wIRA provides a feasible and non-invasive treatment for sacroiliitis. To our knowledge, wIRA has been rarely applied to sacroiliitis, though it has extensive application in skin trauma, burn, thrombosis-related skin ulcer, chronic venous stasis ulcer, bedsores, and other acute and chronic wounds [3]. The benefits of infrared illumination have been widely recognized, but the absorption of radiation at specific wavelength in the infrared region will lead to excessive heating of the skin surface [23]. In contrast, wIRA increases local skin temperature without overheating the skin tissues, which is conducive to tissue perfusion, oxidative metabolism, and disease improvement [24, 25]. wIRA utilizes water-filtering layer to remove the ultraviolet radiation and most radiation in the far-infrared region, while preserving the visible band and infrared A radiation. By making full use of the radiation at a wavelength of 560–1400 nm, which is of the highest therapeutic value and absorbed most by the human tissues, wIRA can reach about 7 cm under the skin. At this depth, wIRA activates the immune system of the lesions and enhances mitochondrial oxygen utilization. Moreover, there will be an increase in oxygen partial pressure of the tissues and enhancement of enzymatic reactions implicating the catalase and superoxide dismutase. In the meantime, the activation of the immune system will facilitate clearing of immunocomplexes and reduce the metabolic products. The pain will be relieved by reducing tonus of the sympathetic nerve and stimulation from the dolorific inflammatory factors and by regulating the pain receptors. Furthermore, oxygen partial pressure of the local inflammatory tissues can significantly affect the VEGF concentration and the density of its receptor, thus improving local blood flow and reducing synovial inflammation and pannus formation [26–28]. In the present study, RI of sacroiliitis increased significantly from 0.53 ± 0.05 to 0.85 ± 0.04 (*P* < 0.001) after the wIRA treatment. But once the treatment stopped, RI decreased from 0.85 ± 0.04 to 0.63 ± 0.04 (*P* < 0.001), indicating that the development of local joint inflammation is driven by systemic and local factors. wIRA treatment alone probably fails to improve the prognosis, as evidenced by the CRP level. Besides, there was a downward trend of serum VEGF levels after wIRA treatment than before, but not significantly. From this, we could infer that local physical therapy was unable to change the systemic inflammatory status. Nevertheless, pain VAS and morning stiffness VAS scores improved significantly in AS patients after wIRA treatment, with a reduction in BASDAI. The treatment efficacy was much more pronounced than the simple use of NSAIDs (*P* < 0.001). wIRA treatment improved the patients’ life quality and compliance in this study.

The present study was limited in the following aspects. Synovial angiogenesis in sacroiliitis in AS patients leads to an increase in venous blood flow. Anatomically, the arteries and veins in the SIJ are very close to each other, which may cause interference to the measurement of blood flow spectrum, resulting in misjudgment. The use of antirheumatic drugs [29] and MTX [30] may inhibit vascular endothelial cell proliferation or angiogenesis, thus affecting the serum VEGF level.

## Conclusions

Finally, wIRA treatment can effectively relieve local inflammation of sacroiliitis in AS, reducing pain and clinical symptoms. Theoretically, there may be less need for NSAIDs, and wIRA treatment may be more appropriate for patients contraindicated for NSAIDs, as an important adjuvant therapy for sacroiliitis.

## Data Availability

The datasets used and analyzed during the current study are available from the corresponding author on reasonable request.

## Abbreviations

ANOVA: Analysis of variance
AS: Ankylosing spondylitis
BASDAI: Bath ankylosing spondylitis disease activity index
CRP: C-reactive protein
HIF-1α: Hypoxia-inducible factor-1α
MRI: Magnetic resonance imaging
MTX: Methotrexate
RI: Resistance index
SIJ: Sacroiliac joint
TNF-α: Tumor necrosis factor-α
VAS: Visual analogue scale
VEGF: Vascular endothelial growth factor
wIRA: Water-filtered infrared A
